# Recurrent Peritonsillar Abscess in Post-tonsillectomy Patient

**DOI:** 10.7759/cureus.22271

**Published:** 2022-02-16

**Authors:** Jacqueline Mirza, Skyler Coetzee, Miguel Belaunzaran, Robert W Trenschel, Tatyana Borisiak

**Affiliations:** 1 Medical School, Dr. Kiran C. Patel College of Allopathic Medicine, Nova Southeastern University, Davie, USA; 2 Internal Medicine, Comprehensive Care Group of South Florida, Plantation, USA

**Keywords:** palatine tonsil, tonsillectomy, pta, tonsil scan, peritonsillar abscess

## Abstract

Peritonsillar abscess (PTA) is a common deep tissue infection of the head and neck. In the literature, most cases demonstrate PTA following acute tonsillitis; however, less documented are cases arising in post-tonsillectomy patients. Here, we report a 45-year-old woman with a history of tonsillectomy 16 years prior, who presented to the emergency department with signs and symptoms consistent with PTA, including sore throat and the presence of a right-sided abscess in the posterolateral oropharynx with apparent pus. The patient reported three previous episodes of right-sided PTA, all of which were addressed via drainage and antibiotic treatment. This episode was treated similarly; cultures from the abscess revealed no growth of organisms. The patient was started on a short course of clindamycin and discharged to follow-up with her primary care physician. Several theories for the etiology of PTA development in post-tonsillectomy patients exist. One theory suggests that PTA may develop in this group of patients due to imperfect margins during the initial surgery, with residual tonsil tissue serving as a nidus for abscess development. Other theories suggest that a congenital fistula may exist in these patients, which, when occluded by scar tissue following a tonsillectomy, may lead to PTA development. Similarly, occlusion of minor salivary ducts has also been suggested to play a role in this unique pathophysiology. Overall, documenting rare cases of PTA development in post-tonsillectomy patients serves as a means of better understanding the complicated etiology behind PTA development and may be able to guide treatment in the future.

## Introduction

Peritonsillar abscess (PTA) is the most common infection of the deep tissues involving the head and neck, with an annual incidence of 41 out of 100,000 persons, and the most commonly affected age group being between 20 and 40 [1-3]. PTA is diagnosed clinically, with one distinguishing feature being inferomedial displacement of the tonsil, often causing displacement of the uvula [1]. Additional symptoms will typically include fever, pharyngitis, trismus, dysphagia, and a muffled, sometimes called “hot potato,” voice [2]. Anatomically, the palatine tonsils, the site of infection in PTA, lie between the palatoglossal arch anteriorly and palatopharyngeal arch posteriorly on the lateral walls of the oropharynx. The surface of each tonsil consists of crypts surrounded by a capsule and a neighboring constrictor muscle. Infection in the space between the fibrous capsule and the superior pharyngeal constrictor is the most common origin of PTA [2]. 

Notably, PTA can occur even in the absence of tonsillar tissue following tonsillectomy. There is a paucity of documented cases demonstrating recurrent peritonsillar abscess in a post-tonsillectomy in the current literature, and therefore, protocols describing management for this rare condition are limited. Here, we present a case of recurrent PTA in a patient 16 years post-tonsillectomy.

## Case presentation

A 45-year-old woman presented to our center’s emergency room with a sore throat. The patient reported an adenoidectomy and tonsillectomy 16 years prior to this presentation, and since that time, she has had three previous episodes of right-sided PTA, all of which resolved with abscess drainage and antibiotic treatment. Prior to this visit, her most recent recurrence was six months ago. 

On presentation, subjectively, the patient reported pain that limited her ability to swallow and drink, but not to breathe, and that she was still tolerating oral intake. She further denied a fever, chills, excessive salivation, and hemoptysis. On physical examination, the patient’s right posterior oropharynx was grossly edematous and swollen, with apparent pus on the right side, and a contralaterally deviated uvula. She was afebrile (37℃), tachycardic (103 beats per minute), tachypneic (18 breaths per minute), and hypertensive (146/81 mmHg), with an oxygen saturation of 100% on room air. The patient’s complete blood count (CBC) was significant for lymphocytosis (14.4 × 10^9 cells/μL), with an absolute neutrophil count of 12.32 × 10^9 cells/μL, and absolute monocyte count of 0.88 × 10^9 cells/μL. Her rapid group A streptococcus and severe acute respiratory syndrome coronavirus 2 (SARS-CoV-2) polymerase chain reaction (PCR) were both negative. 

A CT of the neck soft tissue with IV contrast revealed right-sided tonsillitis with a loculated abscess collection measuring 0.7 × 0.8 cm in size. Reactive lymph nodes were also visualized in the neck, with prominence on the right side. There was no evidence of laryngeal mass, and the visualized oral cavity was unremarkable, with no other enhancing masses. 

The following day, the right parapharyngeal space abscess was drained via needle aspiration. Swab culture from the abscess was negative for aerobic and anaerobic organism growth, with few white blood cells (WBCs). The physician initiated antibiotic treatment of clindamycin 300 mg to be taken orally three times daily for 10 days and Toradol 10 mg to be taken as need for three days for pain management, and the patient was discharged.

At outpatient follow-up, four days following discharge, the patient was tolerating oral intake, was afebrile, and her oropharynx was mildly edematous with no apparent blood or pus. She was continuing her antibiotic regimen of clindamycin 300 mg at that time with no plans for further medical workup.

## Discussion

Cases of PTA in patients with a history of acute tonsillitis have been widely documented for decades [4,5]. This case of a patient with a previous tonsillectomy and no prior history of tonsillitis presenting with recurrent, unilateral PTA demonstrates the complexity of this anatomical region and the need for further exploration of this rare etiology of a common condition [5-7]. According to Chung et al., the recurrence of PTA is associated with extra-peritonsillar spread of the abscess (p=0.007) and history of recurrent tonsillitis (p<0.001) [8]. Within these recurrent cases, *Fusobacterium necrophorum* is most commonly implicated. In a retrospective review by the University of Michigan involving 990 PTA patients from which 150 developed recurrence of disease, prevalence of *F. necrophorum* in the recurrent group was 67% vs 13% (p<0.0001).

The mainstay of PTA treatment is drainage, antibiotic therapy, pain control, and adequate hydration. The administration of corticosteroids has been shown to hasten recovery and reduce the severity of symptoms [2]. If concomitant infection is present, treatment usually targets a polymicrobial infection and therefore includes antibiotics covering oral anaerobes and group A streptococcus [2]. 

During a tonsillectomy, it is challenging to completely excise all tonsillar tissue [5]. One possible etiology for recurrence of PTA in post-tonsillectomy patients is trace tonsillar tissue remaining due to imperfect margins post-tonsillectomy. The peritonsillar space can then become trapped under the hypertrophied remnant tissue, creating a nidus of infection [5,9]. However, even in the absence of tonsillar tissue, several etiologies for peritonsillar abscess have been hypothesized. Roos and Lind described a similar case in a 35-year-old post-tonsillectomy patient who developed a PTA with a culture that revealed growth of *Streptococcus milleri* and *Bacteroides* [7]. 

An alternative theory for the development of PTA in post-tonsillectomy patients is fistula formation [5]. Tonsils are embryologically derived from the second internal pharyngeal pouch, and if the membrane between the pouch and cleft ruptures during development, a fistula may form between tonsillar tissue and the superior constrictor muscle. This congenital fistula may become occluded by scarring post-tonsillectomy, which can impair drainage of infection, predisposing toward recurrent PTA. Therefore, tonsillectomy presents a unique risk to patients with this congenital anomaly [9]. Fistulae between the tonsil and sternocleidomastoid have also been described. Apart from fistulae, second branchial cleft anomalies can also present as sinuses and cysts. Infection of a tonsillar fossa cyst can present similarly to tonsillar abscess [9].

Other theories point to the role that minor salivary glands, Weber glands, play in the formation of PTA, as these glands are just superior to the tonsil and connected by a duct to the tonsillar surface. Weber glands, which are also located throughout the oropharynx, including the tonsils and peritonsillar space, present a possible source of suppuration [5,9]. Obstruction of these glands and ducts may contribute to PTA formation [2]. PTA can also occur concomitantly with parapharyngeal abscess. In a series of 63 patients with parapharyngeal abscess, 33 (52%) patients also had PTA [10]. Finally, the possibility of a parapharyngeal mass, abscess, or tumor must be investigated as they can present similarly to tonsillar abscess, as several cases have been documented [9].

## Conclusions

PTA is the most common soft tissue infection of the deep neck. Therefore, given its prevalence, a complete understanding of all underlying etiologies contributing to this condition is imperative. Here, we report a rare case of recurrent peritonsillar abscess in a post-tonsillectomy patient, having received multiple courses of successful drainage and treatment with antibiotic therapy, and no plan for further medical workup to elucidate why her symptoms recur. The consequence of an incomplete understanding of PTA development, even with non-traditional presentations and etiologies, is medical mismanagement and the development of complications, which include PTA recurrence.
